# Supplementary material for: Vitamin D Modified DSS-Induced Colitis in Mice via STING Signaling Pathway
Source: Biology (Basel). 2025 Jun 18;14(6):715. doi: 10.3390/biology14060715 (PMC12190092; doi:10.3390/biology14060715)
Supplement: Supplementary file 1 [file biology-14-00715-s001.zip › Supplementary Materials S1: 16s-report of gut microbiota/Groups/diff_analysis/RF/Bugbase_Groups.RFimportance.pdf]

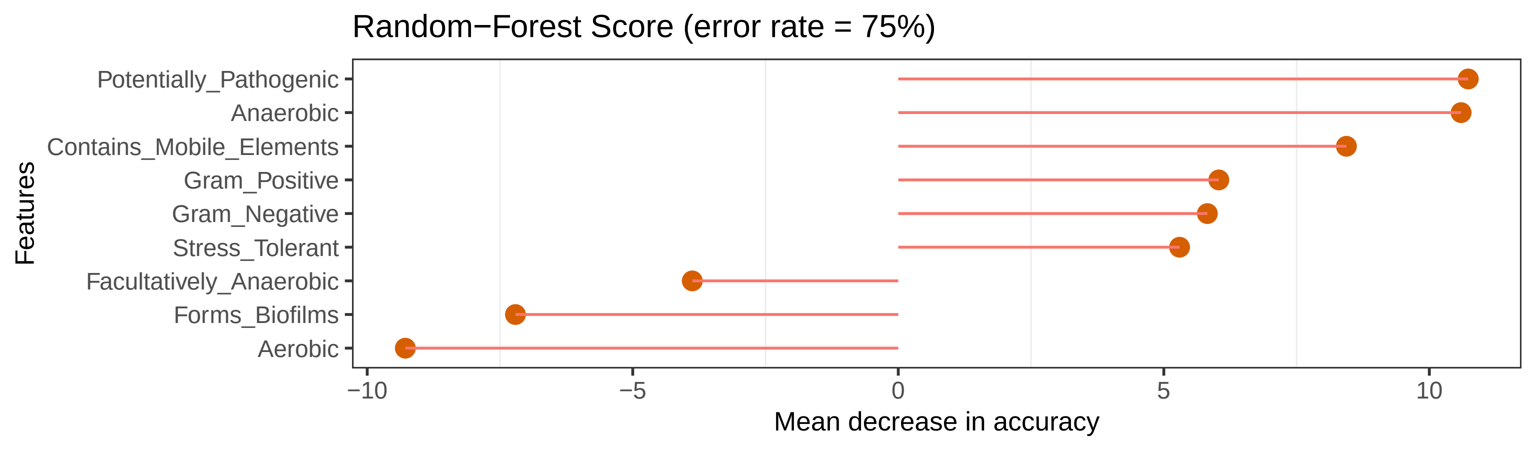

Random−Forest Score (error rate = 75%)
Features
Potentially_Pathogenic
Anaerobic
Contains_Mobile_Elements
Gram_Positive
Gram_Negative
Stress_Tolerant
Facultatively_Anaerobic
Forms_Biofilms
Aerobic
−10
−5
0
5
10
Mean decrease in accuracy
